# Applications of Nanomaterials in Leishmaniasis: A Focus on Recent Advances and Challenges

**DOI:** 10.3390/nano9121749

**Published:** 2019-12-09

**Authors:** Kiran Saleem, Zainab Khursheed, Christophe Hano, Iram Anjum, Sumaira Anjum

**Affiliations:** 1 Department of Biotechnology, Kinnaird College for Women, Lahore 54000, Pakistan; kiran.saleem1196@gmail.com (K.S.); zainabkhursheed777@gmail.com (Z.K.); iram.anjum@kinnaird.edu.pk (I.A.); 2 Laboratoire de Biologie des Ligneux et des Grandes Cultures, INRA USC1328/Université d’Orléans, Chartres 28000, France; christophe.hano@univ-orleans.fr

**Keywords:** Leishmania disease, nanotechnology, liposomes, leishmaniasis, nanovaccine, promastigote

## Abstract

Leishmaniasis is a widely distributed protozoan vector-born disease affecting almost 350 million people. Initially, chemotherapeutic drugs were employed for leishmania treatment but they had toxic side effects. Various nanotechnology-based techniques and products have emerged as anti-leishmanial drugs, including liposomes, lipid nano-capsules, metal and metallic oxide nanoparticles, polymeric nanoparticles, nanotubes and nanovaccines, due to their unique properties, such as bioavailability, lowered toxicity, targeted drug delivery, and biodegradability. Many new studies have emerged with nanoparticles serving as promising therapeutic agent for anti-leishmanial disease treatment. Liposomal Amphotericin B (AmB) is one of the successful nano-based drugs with high efficacy and negligible toxicity. A new nanovaccine concept has been studied as a carrier for targeted delivery. This review discusses different nanotechnology-based techniques, materials, and their efficacies in leishmaniasis treatment and their futuristic improvements.

## 1. Introduction

A diverse group of infectious diseases, commonly known as neglected tropical diseases (NTDs) are predominant in the underprivileged parts of the world, especially in developing countries. These diseases are widespread in tropical and subtropical areas due to poor hygiene and insufficient health infrastructures [1]. Currently, more than 20 different types of NTDs are prevalent in 149 countries, affecting approximately 1.4 billion people worldwide [2]. Leishmaniasis, one of the most neglected tropical diseases, is currently affecting around 12 million people worldwide and 350 million people are under the risk of infection in 98 developing countries [3]. Leishmaniasis has recently earned more public attention due to its high infection and morbidity rate. The London declaration on NTDs was made to eliminate Leishmaniasis as a public health problem by 2020 [4].

**Figure 1:**
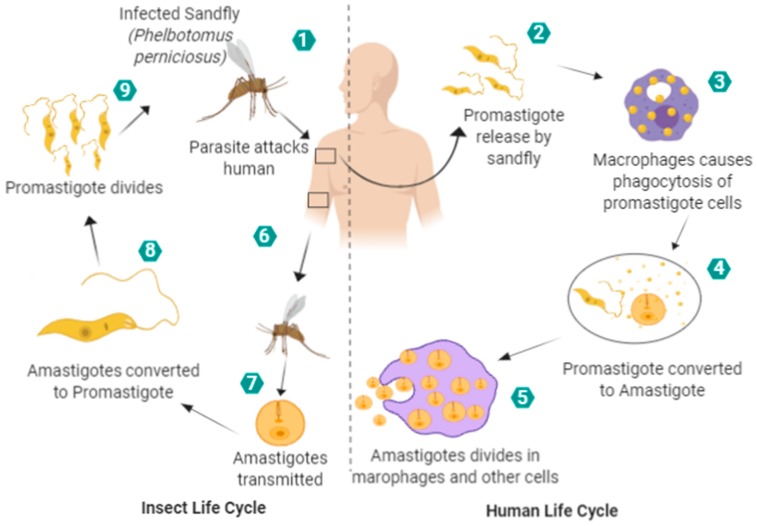
Life cycles of a leishmanial parasite.

Leishmaniasis occurs due to obligate protozoan parasite of the *Leishmania* species [5]. There are almost 51 species of parasites, out of which 21 are pathogenic and cause Leishmaniasis [6]. Some of the species that cause Leishmaniasis includes *L. donovani*, *L. amazonensis*, and *L. aethiopica* etc. Leishmanial parasites exist in two major forms: round and elongated. The round parasite is small and non-motile, while the elongated parasite can move with the help of flagella [7]. Leishmanial transmission occurs when a sand fly sucks blood from an infected individual (human or animal) (Figure 1) [8]. The parasite transformation occurs as it changes from the amastigote stage to the promastigote stage, taking about 4–25 days [9]. The disease results in the development of ulcers and also affects other bodily organs [10]. Leishmaniasis exists in three major forms, namely as mucosal leishmaniasis (ML), cutaneous leishmaniasis (CL), and visceral leishmaniasis (VL). In ML, the symptoms take more time to appear, approximately 1–5 years. The symptoms include runny nose, ulcers formation, breathing problems and nose bleeding [11]. In CL, the symptoms appear few weeks after the person is bitten by sand fly [12] and in the most common type, VL symptoms appear in about 2–6 months, and include weakness, weight loss, fever, enlarged spleen, liver enlargement, lesions, and swollen lymph nodes [13]. Among endemic regions of the world, 0.2–0.4 and 0.7–1.2 million cases of VL and CL have been reported, respectively. Approximately 75% of the global estimated prevalence of CL has been reported among certain countries, for example, in Algeria, Afghanistan, Colombia, Syria, Brazil, Iran, Ethiopia, Costa Rica, North Sudan, and Peru, while more than 90% of VL cases have been reported in Bangladesh, India, South Sudan, Ethiopia, and Brazil [3,14,15].

The leishmanial parasite has ability to take control of the immune system of the affected individuals, which enables the disease condition to persist for a long time and develop into a chronic infection [16]. Basically, the parasite imbalances the host immunity due to its uncontrolled growth inside the macrophages, leading to the eradication of innate, as well adaptive, immunity of the host. There are two ways by which leishmanial parasite manipulates the immune system; by one way the parasites hide in long-lived macrophage cells surviving hostile conditions [17]. The other way is that the parasite mediates a cell signaling pathway in macrophages which inhibits T-helper cells’ (Th2) cytokine responses, specifically interleukins, IL-5, IL-4, and IL-13, leading to downregulation of the protective immune response [18]. Hence, the parasite has the ability to switch between a pro-inflammatory Th1-type healing response to an anti-inflammatory Th2-type non-healing response, which prioritizes their survival and growth inside the macrophages [19]. Additionally, the parasite has also the ability to inhibit the intracellular leishmanicidal activity by decreasing the production of reactive oxygen species, nitric oxide, and pro-inflammatory cytokines leading for their better growth and survival by reduced proliferation of CD4+ and CD8+ T cells, which eventually leads to an enhanced Th2 response [20,21]. Furthermore, several co-inhibitory molecules, such as CTLA-4, PD-L1, CD200, and Tim-3, have shifted the balance of the immune system towards the non-healing Th2 response [19].

The lack of knowledge regarding the Th1 to Th2 cell shift in the host immunological response is due to the unidentified host or parasitic factors that contribute to the severe pathology of Leishmaniasis. Due to the lack of demarcated entities for protective immunity of the host, the generation of vaccines for the parasite has been a difficult task for researchers. Several *leishmania* vaccine candidates have been developed and evaluated in native and recombinant form, like gp63, gp46, TSA, PSA2, LACK, LmsT1, Leish111f, and m2, to kill parasites. However, none of them have shown any outcomes towards prophylaxis [22,23]. Hence, the lack of prophylactic measures has been a concern in the elimination of this NTD.

Although the control measures for the elimination of Leishmaniasis are limited, yet two strategies have been applied, such as classical therapeutics interventions and vector management through insecticides for the control of the *leishmania* parasite in disease-endemic regions [24]. The currently available therapeutic interventions are not effective antileishmanial drugs, besides their enhanced number of cases with relapse and repercussions, have made the current situation critical for the elimination of leishmaniasis [25,26]. Thus, the search for safer, more efficient, innovative, cost-effective therapies is urgently needed for treating Leishmaniasis.

During the last decade, nanotechnology-based drug delivery systems have been used to enhance the performance of drugs in treating several diseases. Combined use of a nanocarrier system with the antileishmanial drugs is a new and promising approach as these nanocarriers can penetrate the macrophages’ cells and reach the infectious parasite, enabling targeted and efficient delivery [27]. A diverse group of nanocarriers also serve as a method of enhancing efficacy, regulating pharmacokinetics, and reduced drug toxicity with sustained release of the drug. Nanotechnology has enabled advancements in pharmacology by providing treatment to various forms of leishmaniasis by targeted delivery of drugs [28,29]. Various nanotherapeutics have been approved by the Food and Drug Administration (FDA) and are currently available for clinical use [30]. Therefore, in this review we attempt to comprehensively compile the various recent reports on nanotechnology-based approaches for the treatment of Leishmaniasis with emphasis on the utilization of various nanosized techniques and nano-drug conjugation systems.

## 2. Conventional Treatment Strategies against Leishmaniasis and Their Limitations

With research advancements, several diagnostic and therapeutic lines have been developed against leishmaniasis. Among all forms of leishmaniasis, VL could be fatal as it affects the organs if not treated properly within two years [29,31]. The diagnosis of leishmaniasis is usually done by examination of the tissue of lesions under microscope. Several molecular-based diagnostic techniques, such as polymerase chain reaction (PCR) and real-time PCR, have also been developed with high sensitivity and specificity [32,33]. However, the main issue associated with these molecular tools is the lack of their availability in health centers of underdeveloped countries. The other method includes the culturing of parasites in different media, such as Novy-MCNeal-Nicolle [34].

The treatment of leishmanial disease has always been a challenge for researchers. In the early 1950s, sodium stibogluconate and meglumine antimoniate were a few of the first anti-leishmanial drugs, but they had many side effects associated with their intake [35]. With advancement in technology and studies, now, there are many therapeutic drugs available for leishmaniasis treatment, including pentamidine, miltefosine, paromomycin, amphotericin B, and its lipid formulations [36,37,38]. The typical chemical compounds employed for anti-leishmanial drug development includes antimony sulfide, doxorubicin, quillaja, saponin, and phosphatidylserine. Although patients with compromised immune systems, cardiac diseases, and organ transplantation cannot be given drugs like pentavalent antimonial [39], it has been recently found that amphotericin B (AmB) is the most effective drug for anti-leishmanial activity. AmB was initially used as an antifungal compound consisting of deoxycholate salt [40]. AmB with encapsulation of liposome has been found to be more effective than AmB alone. Liposmal AmB is less toxic than AmB alone, although it becomes costly [41]. Miltefosine (Impavido) is the only orally administered anti-visceral leishmanial drug. However, the issue associated with miltefosine is that it cannot be used in pregnant and feeding women because it can harm the developing fetus in the womb [42]. A new drug, Humatin has been developed recently with similar efficiency as AmB but with a limited number of side effects [43].

Vaccination is another approach that is employed for leishmanial treatment. The convention vaccine for leishmanial immunization made use of the killed parasite as an antigenic component but its efficacy was low [44]. Later, another approach for fighting the leishmania parasite was introduced, known as a peptide vaccine [45]. The approach is based on the utilization of a minimal pathogenic component to generate long-lasting immunity against the deadly parasite. The choice of epitope is very crucial in peptide vaccine development and, therefore, different in vitro and in silco analysis are conducted to determine the immunogenicity of the peptides [46]. The potential peptides are compared and the best epitope candidates are used for vaccine development by combining multiple epitopes. Peptide vaccine is a promising approach for leishmanial treatment but the challenge is that it is degraded very easily in the body by the immune system [47].

In conclusion, all the available antileishmanial treatments have some limitations or side effects associated with them. Chemotherapeutic drugs are expensive and the parasite has developed resistance against them. Clinical mishandling of medicines in a majority of underdeveloped countries has played a key role in the development of widespread resistance against leishmanial disease [48,49]. In addition, to date there is no effective vaccine available on the market to prevent leishmaniasis [50]. Thus, it is very important to develop alternative drugs via adopting novel strategies that can effectively control this fatal disease.

## 3. Nanotechnology: A New Horizon for Treatment of Leishmaniasis

Innovations in interdisciplinary sciences have been moving the translational sciences to the next level for better control of infectious diseases. Nanomedicine (the use of medical applications of nanotechnology for human welfare) is one of the promising fields in this area that has been continuously growing, keeping up hope for highly sensitive diagnostic tools and better drug delivery for various infectious diseases in the near future [51]. As the traditional antileishmanial drugs have low tolerability, long treatment duration, and are difficult to administer, a tremendous upsurge has been observed in the development of novel nano-biopharmaceuticals that can cure leishmaniasis.

**Figure 2:**
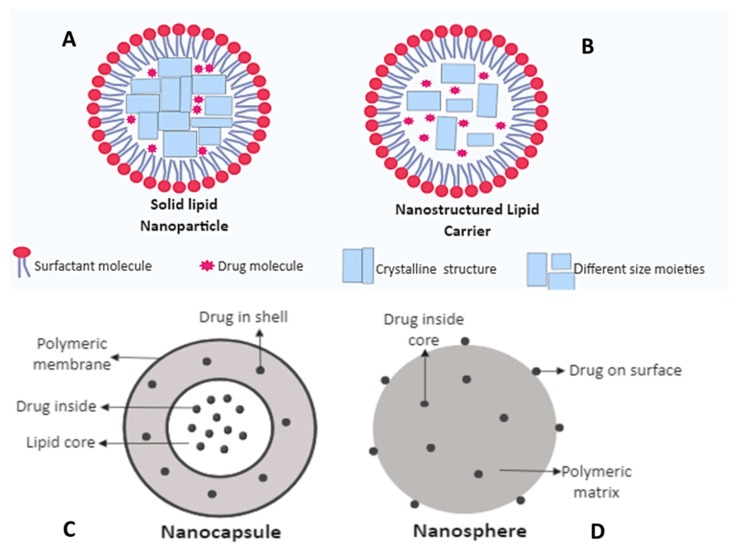
Structure of different nanocarrier systems used for drug delivery. (**A**) Solid lipid nanoparticle (**B**). Nanostructured lipid carrier (**C**). Nanocapsule (**D**). Nanosphere.

The field of nanotechnology has played a vital role in revolutionizing the process of delivering drug in the field of medicine. The nanotechnology employs the use of various drug-loaded nanocarrier systems, such as metallic nanoparticles, liposomes, nanoemulsions, nanosphere, solid-lipid nanoparticles, nanocapsules, polymeric nanoparticles, and nanostructured lipid carriers and nanostructured layered films for efficient drug delivery to the target sites for the treatment of leishmaniasis (Figure 2) [52,53,54,55]. These nanocarrier systems enable targeted delivery, increased bioavailability, and reduced toxicity of drugs [56]. Nanocarriers enclose the drugs that provide targeted delivery and also protect the drug from being metabolized [57].

The absorption and distribution profile of nanocarriers greatly depends on the physicochemical properties, i.e., the size, hydrophobicity, targeting molecule, and their charges. Many processes, like uptake and entry of nanocarriers into cell and their further interaction with immune system, are dependent on the size and charge of the nanocarriers. Anther property is the hydrophobicity, which controls the absorption and distribution of nanocarriers by effecting the immune cells’ interaction, protein interaction, particle clearance, and protein charge [58,59]. The charge is used in binding plasma protein, protein interactions, membrane damage, and in immune cell stimulation [60].

**Figure 3:**
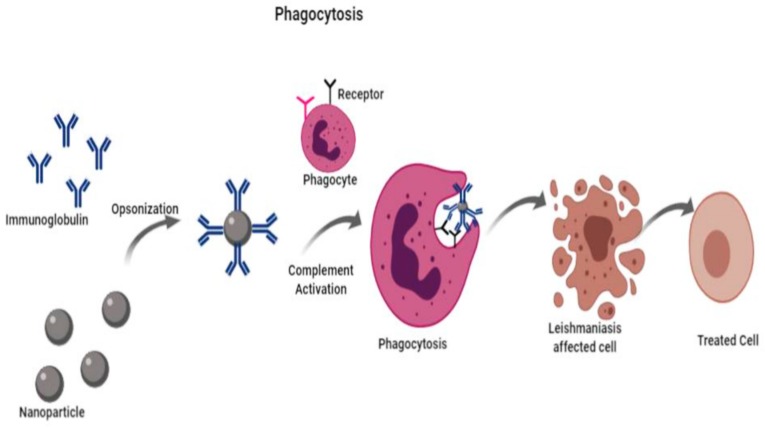
Schematic representation of phagocytosis process for absorption of nanoparticle.

These drug-loaded nanocarriers enter the cell by phagocytosis, a form of endocytosis in which the cell engulfs particles larger than 0.75 µm in diameter. Macrophages, neutrophils, and monocytes are capable of phagocytosis and, therefore, sometimes are referred as “professional phagocytes” [61]. Leishmaniasis is a particularly interesting disease to be treated with drug-loaded nanocarriers since the parasites exclusively infect the highly phagocytic cells known as macrophages. In this way, the macrophages take up the drug-loaded nanocarrier by phagocytosis, where they will directly act on the parasites (Figure 3). This allows the drugs to reach an effective intracellular concentration, along with a reduction in toxicity and dosage of drugs [62,63]. Furthermore, there are two types of vectoring procedure, active and passive vectoring, which could affect the distribution and uptake. In active vectoring, a specific compound is added or attached to the surface of the nanoparticle, whereas passive vectoring is the inherent capacity of cells when they recognize the foreign particles to organisms [61].

## 4. Drug-Loaded Nanocarrier Systems for Treatment of Leishmaniasis

Various nanocarrier systems have been synthesized and used in the controlled drug delivery in treatment of leishmaniasis. Each of these nanocarrier systems have their own advantages and disadvantages, as discussed in Table 1. Among the traditional nanoparticles, the most preferred are liposomes and polymeric nanoparticles as they are easily and rapidly internalized by macrophages in the liver and spleen [64]. The most commonly employed nanocarriers in curing leishmaniasis are liposomes due to its unique properties. They are able to load and deliver both hydrophobic and hydrophilic drugs by surface functionalization, which is used to improve drug targeting. Additionally, the fate of liposomes and the leishmaniasis parasite is the same. The positively-charged liposomes are readily taken in by the macrophages. Since the macrophages can recognize sugar molecules, liposomes are surface functionalized with sugar to improve macrophage targeting. However, liposomes face some limitations, as well. They are not stable. They could result in toxicity because the drug can leak from the liposomes into the blood supply [65].

Nanoemulsions are one of the best drug delivery systems due to their simple preparation, ability to solubilize hydrophobic drugs, physicochemical stability, and easy scale-up [66]. Polymeric nanoparticles are also a widely used nanoparticle system for the treatment of leishmaniasis. They have the properties to overcome some of the drawbacks of liposomes [67]. The have small size, low toxicity, and are cost effective as they can be used to deliver more than one drug. They have the ability to design biodegradable systems and can be surface functionalized. Among the polymeric nanoparticles, poly lactide-co-glycolide (PLGA) is the most commonly employed as it is biodegradable and biocompatible. One important difference between liposomes and polymeric nanoparticles is their stability [53]. Unlike the unstable nature of liposome, polymeric nanoparticles do not face the limitation of drug leakage into the blood supply [68,69].

There are some advanced nanocarrier systems, such as metallic nanoparticles, dendrimers, and carbon-based nanomaterials. They need to be studied well in order to know their advantages and drawbacks. One important advantage of dendrimers is their ability to load more than one drug due to their branched structure, enhancing drug bioavailability [70]. Along with the advantages of nanocarriers as efficient antileishmanial drugs there are also some challenges to overcome. One of the prominent hurdles is the high cost of these nanoformulations. Hence, their commercialization and high scale production is not economically feasible. On account of their economic feasibility, solid-lipid nanoparticles (SLNs) are better because they are made of triglyceride lipids whose production scale up is less expensive then phospholipids [71].

### 4.1. Liposome Nanoparticles

Liposome nanoparticles are nano-sized spherical vesicles that are made up of bilayer phospholipids which provide an aqueous support which serves as a carrier for the adherence of both hydrophilic and lipophilic drugs [79]. Liposome usage has many advantages over conventional drugs: they have increased retention in the body and stay in circulation for a longer period of time [80]. Liposomes have the ability of sustaining the drug release, controlling the release, and reducing the drug dosage and its frequency of dosage [81]. Due to these properties, liposomes are largely employed for the study of leishmaniasis treatment and, hence, are the most anticipated clinical drug. In case of anti-leishmanial treatment, the drugs are encapsulated in a liposomal layer which enables their efficient intracellular delivery to the leishmanial amastigotes. These liposomes have the ability to penetrate the macrophages by the process of phagocytosis and directly deliver the drug to the site of the parasite [82].

The nanosized liposomes are an emerging approach used in many disease treatments, particularly for the delivery the chemotherapy drugs. The liposomes provide enhanced pharmacokinetic properties along with target precision which provides a major advantage [83]. The best example of liposome is AmB lipsome, a formulation of AmB liposome, which reduces the side effects of amphotericin B [84]. In the AmB formulation there are different types, such as AmB colloidal solution, AmB liposome, and AmB lipid network, amongst which the liposomal AmB has been proven to be most effective [85]. According to one study, the formulation of the liposome can overcome the disadvantages of conventional drugs. The different formulations, including miltefosine, paromomycin, and meglumine antimoniate, have been developed against leishmania by the process of freeze drying. The liposomal drug was administered by subcutaneous injection in mice and the efficiency was studied, which turned out to be 90% [86].

In another study it has been reported that the macrophages have receptors on their surface that play a role in the control of cellular functions, such as recognition, activation, secretion, and endocytosis [87]. The liposome, incorporated with a ligand, interacts with the receptors of macrophages enabling the uptake of the liposomal content [57]. The liposomes conjugated with mannose and 4-sulphated acetyl galactosamine have been found to be effective against leishmanial activity [88].

### 4.2. Lipid Nano-Capsules

Lipid nano-capsules (LNs) are nanocarriers that range in size from 20 to 100 nm and they mimic the lipoproteins. Lipid nanocapsules consist of a core of lipids and a surfactant membrane surrounding it. It is a hybrid structure made from the use of liposomes and polymeric nanocapsules [89]. LNs are made by use of a solvent-free method which provides the stability and increased bioavailability. The major advantage of LNs is that they deliver the drug on site, minimizing the dosage by many folds and reducing the side effects of toxicity [90]. In one study for the development of LNs, the core was made of hydrophobic olive oil and the outer shell was made of a hydrophilic component, chitosan [91].

The miltefosines are alkylphospholipids that are used in the treatment of leishmaniasis destroy the Ca^2+^ homeostasis of parasite. The formulation of NPs loaded with miltefosine provided enhanced effectiveness against leishmaniasis, which was demonstrated by the injury to the promastigotes. The stability and sustained drug release were also ensured by the LNs. The LN oral drugs have been developed with advancements in technology [92].

### 4.3. Metallic Nanoparticles

Metals have been used in medications since early history. There is a wide range of metallic nanoparticles that are being used for antileishmanial activity providing minimal toxicity and high efficacy [73,93]. The metallic nanoparticles first came into existence in the 1850s. A study was conducted for the treatment of visceral leishmaniasis with iron oxide (Fe_3_O_4_) nanoparticles coated with glycine (peptide), which encapsulated the AmB drug. A 10–15 nm nanoparticle size was used, which enabled the controlled release of AmB, reducing the parasitic content in the spleen of treated subjects [94]. Glycine-coated nanoparticles could be employed further in leishmanial treatments.

Zinc oxide nanoparticles (ZnONPs) are massively produced and used. A study was conducted in which ZnONPs were employed in varying concentrations (0.18, 0.37, 0.75, and 1.5 µg/mL) against the amastigote form of leishmania, *L. donovani*, in vitro culture. The results were analyzed by colorimetric assay which suggested that ZnONPs exerted a cytotoxic effect on the amastigote cells, causing hinderance in their proliferation and suppression of activity of *L. donovani*. The study suggests that ZnONPs could be a cost-effective means against anti-leishmanial drug development [95]. Sumaira, et al. [96] prepared ZnONPs from *Verbena officinalis* and *Verbena tenuisecta* plant leaf extracts. The results suggest that *V. officinalis* had more phenolic content. Both plant ZnONPs were tested for anti-leishmanial activity, where the *V. officinalis* ZnONPs had better activity due to the greater phenolic content and smaller size as compared to *V. tenuisecta*-mediated ZnONPs.

Silver has always been very useful in medications since early times. Silver colloidal solutions were initially used for treating infections; approximately 650 different diseases and illnesses were treated [97,98]. Later, with advancements, nanotechnology helped to develop nanosilver or silver nanoparticles (AgNPs). Various studies have been conducted on the biogenic synthesis of silver AgNPs and their mode of actions in different biomedical applications [99,100]. Antileishmanial activity of AgNPs was checked, obtained from a fungus source, *Fusarium oxysporium*, and evaluated by a group of researchers [91]. The results of the study were promising as AgNPs led to the death of promastigotes enabling its apoptosis. In further studies it was found that the AgNPs release reactive oxygen species (ROS) that cause damage to the membranes of promastigotes. In the case of amastigotes, the AgNPs led to a reduction of infected macrophages. AgNPs had a direct damaging ability against amastigotes [91]. Another AgNP study suggested that the anti-bacterial activities of silver nanoparticles help fight leishmaniasis. In this study, the effects of AgNPs were checked against leishmanial parasite morphology, infectivity, metabolic activity, survival abilities, and proliferation rates. AgNPs led to impairment of morphological characteristics and infectivity rates of the parasite. Additionally, the metabolic activity and proliferation was reduced by 1.5-fold [90]. Overall, the AgNPs could be a new therapeutic source for the treatment of leishmaniasis [90,101].

The use of nanoparticles under ultra-violet (UV) and infrared (IR) light have high toxicity by generating ROS, causing the death of the parasites. In one study, the antileishmanial effects of some nanoparticles, such as AgNPs, gold nanoparticles (AuNPs), titanium dioxide nanoparticles (TiO_2_NPs), ZnONPs, and magnesium oxide nanoparticles (MgONPs) were evaluated on leishmania major parasites [28,102]. Increased antileishmanial activity was observed for AgNPs, followed by AuNPs, TiO_2_NPs, ZnONPs, and MgONPs under UV and IR light conditions as compared to dark. Thus, both of these light-improved antileishmanial properties of these nanoparticles must be considered in future studies [28]. Similarly, in another study, chitosan-derived TiO_2_NPs were used as an effective antileishmanial agent against amastigote and promastigote forms of the parasite. Chitosan-derived TiO_2_NPs were loaded with meglumine antimoniate to enhance the activity of TiO_2_NPs. The activity of the nanoparticles was checked via a UV spectrophotometer. The formulation was found to be effective against amastigote as well as promastigote forms of the parasite [92]. Overall, metallic and metal oxide nanoparticles provide a promising approach for the reduction and treatment of all types of leishmanial activity [92,103].

### 4.4. Polymeric Nanoparticles

These nanoparticles are made from various types of biocompatible and biodegradable colloidal particles. Their size ranges from 10 to 1000 nm [104]. They carry drugs by different approaches, like adsorption, encapsulation, dissolution, entrapment, or by chemically binding the drug on the surface of polymeric nanoparticles (PNPs) [105]. Advanced physicochemical properties of PNPs lead to improved bioavailability, enhanced cellular dynamics, biodegradability, and controlled drug delivery [74].

Polymers are the most widely studied and researched form of carriers used in nanomedicine. It was first used in 1979 for cancer therapy when polyalkylcyanoacrylate nanoparticles were used to adsorb anti-cancer drugs [106]. PNPs include synthetic polymers, such as poly (lactic acid) (PLA), poly (glycolic acid) (PGA), poly (lactide-co-glycolide) (PLGA), poly (caprolactone) (PCL), poly (cyanoacrylate) (PCA), and natural polymers, such as gelatin, albumin, chitosan, and alginate [107,108]. Among these polymers, PLGA has been mostly used in drug delivery and in tissue engineering. PNPs are present in two different forms: nanospheres and nanocapsules, polymeric or reservoir systems, respectively. In nanocapsules, the drug is encapsulated in a cavity surrounded by a polymer membrane, whereas the drug is not confined in a cavity, but it is dispersed uniformly in the case of nanosphere [109,110].

PNPs appear to be a great choice for delivery of drugs and proteins to target cells because of their easy permeation due to their small size, and these polymers can be designed in various molecular designs with many applications [75]. PNPs deliver drug to the targeted site by the following three mechanisms:

1. Through an enzymatic reaction which lead to polymer degradation at targeted site resulting in the release of the drug.
2. Through swelling of the PNP followed by hydration and drug release by diffusion.
3. Through detachment of the drug from the polymer [111].

PNPs are being studied for their use as a drug carrier for the treatment of leishmania. Different types of PNPs are being studied on mouse models, in in vitro studies, to investigate the treatment for leishmania [112,113]. A studied conducted by Roy et al. [76] studied the effect of nano-encapsulated diterpenoid lactone and andrographolide on albino mice. Poly(DL-lactide-co-glycolic acid) nanoparticles and polyvinyl alcohol (PVA) was loaded in a 50:50 ratio. The results showed significant antileishmanial activity on mouse models using 4% PVA on using 1/4 of the pure compound dosage. The authors suggested that this could provide a cost-effective chemotherapy of leishmaniasis.

A recent study was conducted to investigate the efficacy of carbohydrate-functionalized PLGA (poly lactide-co-glycolide) nanospheres for the treatment VL in mice [114]. PLGA nanospheres were prepared by nanoprecipitation and surface functionalized with three different types of carbohydrates, i.e., mannose, mannan, and mannosamine. Co-culturing of these PLGA nanospheres with macrophages led to the activation of immune-modulatory and pro-inflammatory responses in the host, which triggered the killing of parasites. The authors reported that the mannan-functionalized PLGA nanospheres were more effective against VL parasites as compared to the mannose- and mannosamine-functionalized PLGA nanospheres [114].

### 4.5. Solid Lipid Nanoparticles (SLNs) and Nanostructured Lipid Carriers (NLCs)

Solid lipid-based nanoparticles are a relatively new class of nanocarrier. Solid lipid nanoparticles (SLNs) and nanostructured lipid carriers (NLCs) belong to this class and differ from each other based on their matrix [111]. SLNs are nanospheres made from lipids which remain solid at body temperature and are stabilized by emulsifiers [115]. Their size is less than 1000 nm [116]. The have various advantages as they protect the drug against harsh environmental conditions, their large-scale production is easy, using high-pressure homogenization technique, they are biocompatible and biodegradable [117]. However, they have some limitations as well, i.e., SLNs have low drug loading efficacy due to its crystalline structure and there is a chance of drug expulsion during the storage of the crystalline structure and initial burst release can occur [118].

A study revealed that chitosan-coated SLNs carrying AmB were synthesized for chemotherapy of *Leishmania* infections. Their antileishmanial activity showed that the SLNs have a strong effect than formulations of AmBisome and Fungizone, available on the market. Additionally, this study showed SLNs are safer than market products, by evaluating acute toxicity study in mice [77]. In another study, in vivo efficacy of SLN-loaded paromomycin sulfate was investigated against *L. tropica* in a mouse model. It was found that parasite propagation and switching towards the Th1 response was more effectively inhibited by using PM loaded with SLNs as compared to when paromomycin sulfate was used alone [119]. Nanostructured lipid carriers (NLCs) are referred to as the second generation of solid lipid-based nanoparticles. They are the combination of both solids and lipids, unlike SLNs. It does not have a definite crystalline structure, but rather has different sized moieties. NLCs have better loading capacity since they do not have a crystal structure. Thus, drug expulsion and burst release is not faced in the case of NLCs [120].

A recent study was carried out to make a formulation of a veterinary drug named buparvaquone by using NLCs [117,121]. Another study prepares Amphotericin B lipid nanostructured carriers in order to increase the therapeutic efficacy and reduced toxicity of Amphotericin B, which is the only main treatment against leishmaniasis. Different formulations were synthesized and the selection criteria were particle size and particle size distribution. The in vitro release profile of the AMB-loaded NLCs showed 65% drug release within 24 h. The results of the study showed that delivery of AMB through NLCs is preferable over using Amphotericin B alone [122].

### 4.6. Nanotubes

Nanotubes are cylindrical hollow molecules that are synthesized from inorganic and metallic materials. A number of studies have been conducted which prove nanotubes are excellent nanocarriers. Anti-leishmanial efficacy of AmB associated with carbon nanotubes was examined in a study. The authors found this formulation to have better targeted killing of *L. donovani* compared with free AmB [77,123]. Another study developed betulin associated with CNTs as an anti-leishmanial formulation. The study reported better cytotoxicity of the new antilieshmanial formulation compared to the control group [124].

A study used a formulation of linked AmB, an antileishmanial drug, with functionalized carbon nanotubes (f-CNTs) to lessen the drug-induced toxicity. This formulation was able to inhibit parasite growth more effectively than AmB. This drug carrier improves the drug efficacy. Additionally, there was toxicity observed in the kidneys or livers of mice [125]. The use of carbon nanotubes in drug delivery have not been designed for humans yet, but are in the preclinical stage.

## 5. Nanovaccines: An Emerging Approach of Nanotechnology for Combating Leishmaniasis

To date, chemotherapeutic drugs act as the main treatment of leishmania, including amphotericin B, paromomycin, fluconazole, antimony-containing compounds, and pentamidine. However, these therapeutic drugs have their own limitations, such as toxicity, longer regimens, low efficacy, and drug resistance [24]. Other therapies, which could be effective for eradicating leishmania, are vaccine based. There are two types of leishmanial vaccines being prepared: first-generation and second-generation. First-generation leishmanial vaccines are comprised of live vaccine while second-generation vaccines are made using recombinant technology [126,127]. To date, there is no licensed vaccine for leishmaniasis. Three types of leishmanial vaccines, Leish-F1, F2, and F3, designed at the Infectious Disease Research Institute (IDRI) are in clinical trials [128]. These are formulated on the basis of the selective antigen epitope properties of leishmania. Recombinant leishmania vaccines are also being investigated at the Sabin Vaccine Institute [50].

With the advent of nanotechnology, there is now a new approach of synthesizing vaccines using nanoparticles as carriers of antigen preparation. Nanoparticles could provide a safe, efficacious, and efficient delivery system for vaccines. According to one study, solid lipid nanoparticles can serve as an efficient tool to synthesize leishmanial vaccine [129]. Delivering antigens and adjuvants using nanoparticles have different purposes:

1. To help increase uptake by of the antigen, loaded in nanoparticles, by the antigen-presenting cells [130].
2. To activate a stronger immune effect as a simultaneous delivery of the antigen by different NPs to the same APC, activating the immune response strongly as compared to the free antigen and adjuvant [131].
3. To activate Th1-type immune response [132].

In 2005, a group of researchers prepared a nanovaccine by loading recombinant *Leishmania* superoxide dismutase in a chitosan nanoparticle using the ionotropic gelation method in mice. The study assesses the loading efficacy and size of nanoparticles loaded with SODB1. The results showed higher cell-mediated immune response and higher IgG2a levels, on using stable chitosan nanoparticles, which could be used as a nanovaccine for leishmaniasis [133]. Another study uses nanoliposomes used as the nanocarrier for soluble Leishmania antigens (SLA). Results showed that parasites decreased in the footpad and spleen of a mouse model injected with this formulation when compared with the control group [134].

Although there is no nanovaccine commercially available for leishmaniasis, studies have led to higher efficacy by using nanoparticles as vaccine carriers and as adjuvants to form nanovaccines, which could be potentially decrease the number of leishmaniasis cases.

## 6. Conclusions and Future Outlooks

Despite several treatment options, there is not even a single efficient option that would effectively control the incidence of leishmaniasis. The drugs available for treating leishmaniasis face many side effects, such as high cost, toxicity, and resistance to parasites. Various studies are being conducted to enable the use of nanotechnology for devising nanomedicines and nanovaccines for treating leishmaniasis [44,51,108,135]. Various nanomaterials are being studied for the development of safe and cost-effective drugs for treating leishmania. Many studies have revealed that the potential efficient agents for leishmanial treatment could be liposomes, PLGA nanoparticles, carbon nanotubes, and SLNs that enhance the parasite-targeted drug delivery.

Nanovaccines are a relatively new concept in treating leishmania; although no vaccine is yet available, but studies are on-going to find efficient nanovaccines. Despite many studies that have been conducted to find nanotechnology-based efficient drugs for leishmaniasis, they are all still in the preclinical stage, except for one liposomal drug (AmB) which is commercially available [136]. The commercial aspects of nanomedicines are a major concern for researchers. Any drug delivery system’s most desired characteristic is its commercial feasibility. The cost of drugs can affect the resources and scale up of drug development. AmB is currently the most cost-effective drug in leishmaniasis treatment. The other nanoparticle-based drug delivery strategies are under the process of development and trials only, and their production cost has not yet been analyzed [137]. There is, however, need for advanced studies and research to develop effective drugs with low cost against leishmania disease.

## Figures and Tables

**Table 1:** Advantages and limitations of nanocarrier systems.

Nanocarrier	Advantages	Limitations	References
Liposomes	Ability to carry either, hydrophilic or hydrophobic drugs, biocompatible, biodegradable, stable, possibility of surface functionalization	Toxic because the drug can be leaked or displaced into the blood stream; High production cost	[72]
Polymeric nanoparticles	Biocompatible, low toxicity, biodegradable, cost-effectives, possible surface functionalization, avoids leakage of the drug	Difficult to scale up	[73,74]
Solid lipid nanoparticles (SLNs)	Protect drug against harsh environmental conditions, easy scale up, biocompatible	Low drug-loading efficacy due to its crystalline structure, there is a chance of drug expulsion during the storage of the crystalline structure and initial burst release can occur	[75,76]
Nanoemulsions	Stable, Carry both hydrophobic and lipophilic drugs	Toxicity of surfactants	[77]
Metallic nanoparticles	Antibacterial, Antifungal properties, Stable, Uniform structure	Toxicity	[78]
